# Psychological distress and academic self-efficacy of nursing undergraduates under the normalization of COVID-19: multiple mediating roles of social support and mindfulness

**DOI:** 10.1186/s12909-023-04288-z

**Published:** 2023-05-17

**Authors:** Ting Xu, Pingting Zhu, Qiaoying Ji, Wen Wang, Meiyan Qian, Guanghui Shi

**Affiliations:** School of Nursing and School of Public Health, Yangzhou University, 136 Jiangyang Middle Road, Yangzhou, 225009 China

**Keywords:** COVID-19 epidemic, Academic self-efficacy, Psychological distress, Social support, Mindfulness

## Abstract

**Background:**

Nursing undergraduates’ academic self-efficacy is a significant factor in determining their learning motivation, cognition, and emotions. It has a significant impact on improving academic performance and achieving learning goals.

**Methods:**

To explore the mechanism of psychological distress affecting the academic self-efficacy of nursing undergraduates, the generalized anxiety disorder scale-7, patient health questionnaire-9, academic self-efficacy scale, perceived social support scale and mindful attention awareness scale were conducted.

**Results:**

Model fitness indexes of the structural equation model is good (CMIN/DF = 1.404, RMSEA = 0.042, GFI = 0.977, IFI = 0.977, TLI = 0.954, CFI = 0.975, NFI = 0.923). Structural equation model analysis showed that social support and mindfulness were the mediating variables of psychological distress on academic self-efficacy. Mediating variables accounted for 44% of the total effect value (− 0.3) with a value of − 0.132. Three paths were verified: psychological distress indirectly affected academic self-efficacy through social support (− 0.064); psychological distress indirectly affected academic self-efficacy through mindfulness (− 0.053); psychological distress indirectly affected academic self-efficacy through social support and mindfulness (− 0.015).

**Conclusions:**

Social support and mindfulness play significant mediating roles in the effect of psychological distress on academic self-efficacy, and the chain mediating role of social support and mindfulness is also significant. Educators may mitigate the impact of psychological distress on academic self-efficacy by enhancing students’ social support and mindfulness.

## Introduction

In recent years, the effect of learning thoughts and beliefs on students’ academic performance has attracted more and more attention from educational researchers [1]. Self-efficacy is one of the most important personal beliefs. Educational researchers agree that academic self-efficacy is an important factor in determining students’ learning motivation, cognition and emotion, and has a significant impact on students’ firm belief, learning and achievement goals [2–4]. Academic self-efficacy has also been regarded as one of the predictors of students’ academic performance [5–7]. Nursing undergraduates have always been faced with long cycles of learning, heavy tasks of learning and frequent examinations [8]. These academic pressures require them to have a high level of academic self-efficacy to complete their learning tasks and goals. However, the COVID-19 pandemic has reduced the academic self-efficacy of college students, and junior nursing college students reported that their academic self-efficacy was only at a moderate level [9, 10]. Low academic self-efficacy will lead to the weakening of students’ learning motivation, learning ability and learning effect, thus hindering their reserve of professional knowledge and increasing the sense of uncertainty in clinical work [4, 11, 12]. Students with high self-efficacy tend to solve problems when they encounter difficulties, which is more conducive to their academic achievement and paves the way for their careers [13, 14].

Psychological distress is one of the most common problems among medical students [15, 16]. Low mood, depression, irritability, and anxiety are all common symptoms of psychological distress. In the past COVID-19 pandemic, the risk of COVID-19 virus infection closed campus management, the cancellation of recreational activities, the interruption of clinical practice, and heavy online learning tasks made the psychological distress of medical students particularly prominent [17–19]. Nursing undergraduates are among the medical students affected. These psychological problems have a negative impact on nursing undergraduates’ academic self-efficacy, which is reflected in the decline of their learning enthusiasm and efficiency [20–22]. It is very important for nursing undergraduates to accumulate professional knowledge and practical skills before entering nursing work, which directly affects their future ability of nursing [19, 23]. Therefore, it is very important to pay attention to the influence of nursing undergraduates’ psychological distress on academic self-efficacy.

Fortunately, the multi-factor theory of psychological stress provides a solid theoretical basis for solving the effect of psychological distress on the academic self-efficacy of nursing undergraduates [24]. This theory holds that under the influence of stressors, individuals can relieve the negative effects of stressors on individual physiology and psychology by mobilizing their internal and external available resources.

As one of the internal resources of individuals, mindfulness can guide individuals to pay attention to their current emotional state and physiological health, to alleviate the negative effects caused by stressors [25]. The mindfulness-based coping model suggests that mindfulness can help individuals reevaluate negative or threatening events, relieve psychological distress caused by stress, anxiety, and depression, help individuals regain confidence, and enhance their ability to adapt to various environmental changes [26]. A high level of mindfulness is also beneficial to relieve students’ psychological distress, which will improve their concentration ability and self-confidence, make them more focused on learning and firm goals, and then improve learning efficiency and achieve their ideals [27, 28]. In addition, as one of the most common and effective external resources, social support can encourage and support individuals under the action of stressors, and enhance their confidence and ability to cope with difficulties [15, 29]. Students with more social support have more confidence in achieving their learning goals. Therefore, it can be speculated that the level of mindfulness and social support of nursing undergraduates play a mediating role in the effect of psychological distress on academic self-efficacy.

**Fig. 1:**
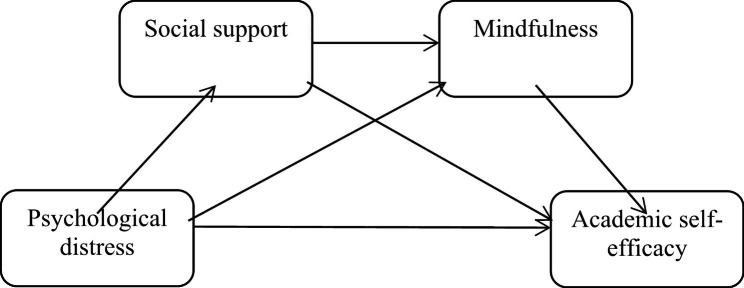
The conceptual model

During the COVID-19 pandemic, a large number of studies have focused on the psychological health and academic self-efficacy of nursing undergraduates. However, few studies have explored the current status of academic self-efficacy among nursing undergraduates under the normalization of COVID-19. Therefore, based on the multi-factor theory of psychological stress, this study first wanted to explore the current status of academic self-efficacy and its related influencing factors (psychological distress, mindfulness and social support) among nursing undergraduates after the COVID-19 pandemic. The second purpose was to verify the mediating role of mindfulness and social support in the relationship between psychological distress and academic self-efficacy by constructing and analyzing the structural equation model. In summary, this study proposed the following hypotheses (See Fig. 1):

H1: Social support of nursing undergraduates is the mediating factor that regulates the effect of psychological distress on academic self-efficacy;

H2: Mindfulness of nursing undergraduates is the mediating factor that regulates the effect of psychological distress on academic self-efficacy;

H3: Psychological distress of nursing undergraduates affects academic self-efficacy through the chain mediation of social support and mindfulness.

## Materials and methods

### Study design and setting

This study was a web-based cross-sectional survey from April to May 2022, which invited all nursing undergraduates from their freshman to senior years in two nursing schools of a university in Jiangsu Province, China (web sites for online questionnaires: the ‘Questionnaire Star’, https://www.wjx.cn). Nursing undergraduates at this stage have just finished the protective isolation of COVID-19 and gradually returned to offline learning.

### Study participants

All students over the age of 18 who had the following characteristics were excluded: a past mental or psychiatric illness, had participated in a similar study in the past three months, and were currently infected or isolated. According to Nevitt and Hancock, the sample size required for structural equation modelling should exceed at least 200. In addition, considering the 10% non-response rate of the questionnaire, this study needs to investigate at least 220 students.

### Data collection

A pre-survey study was conducted to further improve the quality of the questionnaire before the formal survey. A total of 40 nursing undergraduates (10 students in each grade) were randomly selected to participate in the pre-survey. The normal questionnaires were revised according to the feedback of the pre-survey to ensure the rationality and comprehensibility of the questionnaire. The questionnaire survey was published online by the teacher in their WeChat group of classes before the start of the online courses. The students voluntarily filled in the questionnaire through mobile phones or personal computers. In addition, before filling out the questionnaire, students are informed of the purpose of this study and the content of the questionnaire. Students need to sign the electronic informed consent form before filling in the questionnaire, and they can withdraw at any time in the process of research. All questionnaires are submitted anonymously and all data are kept confidential. Finally, a total of 253 questionnaires were collected and 26 invalid questionnaires were excluded, leaving 227 valid questionnaires.

### Data collection tool

#### Socio-demographic characteristics

The socio-demographic survey collected information on the gender, age, grade, place of origin, monthly household income, academic performance, and professional preferences of nursing undergraduates.

#### Academic self-efficacy scale

The scale was compiled by Liang, including college students’ academic ability self-efficacy and academic behaviour self-efficacy, each dimension has 11 questions [30]. Using Likert 5 rating method, ‘1’ means completely inconsistent, ‘5’ means completely consistent, and the higher the score, the higher the individual learning self-efficacy. The questionnaire has been widely used among college students and demonstrated good reliability [31]. The Cronbach’s alpha of this scale was 0.85 in Lian et al.’s study and 0.89 in this study, and Cronbach’s alpha of academic ability self-efficacy and academic behaviour self-efficacy were 0.912 and 0.689, respectively.

#### Generalized anxiety disorder Scale-7 (GAD-7)

The scale was compiled by Spitzer et al. and translated into Chinese by Li et al. with a total of 7 items (0 ~ 3 points for each item) [32, 33]. The higher the score, the more severe the symptoms of anxiety. According to the score, people were divided into no anxiety (0 ~ 4 points), mild anxiety (5 ~ 9 points), moderate anxiety (10 ~ 14 points), and severe anxiety (≥ 15 points). The scale has been widely used among college students and demonstrated good reliability [34]. The Cronbach’s alpha of this scale was 0.898 in Li et al.’s study and 0.91 in this study.

#### Patient health questionnaire-9 (PHQ-9)

The scale was developed by Spitzer et al. based on the symptomatic criteria for depression in the fourth edition of the Diagnostic and Statistical Manual of Mental Disorders [32]. The scale can measure depression-related symptoms over the past two weeks and can be used to screen for depression and assess the severity of symptoms. PHQ-9 contains 9 items (0 ~ 3 points for each item). The higher the score, the more severe the symptoms of depression. According to the score, individuals were divided into no depression (0 ~ 4 points), mild depression (5 ~ 9 points), moderate depression (10 ~ 14 points), moderate to severe depression (15 ~ 19 points), and severe depression (20 ~ 27 points). The scale has been widely used among college students and demonstrated good reliability [35]. The Cronbach’s alpha of the scale was 0.89 in Beswick et al.’s study and 0.913 in this study [36].

#### Multidimensional scale of perceived social support (MPSSS)

The scale was compiled by Zimet et al. and translated into Chinese by Zhou et al. The scale contains 12 items, which are divided into three dimensions: family support (3-4-8-11 items), friend support (6-7-9-12 items) and other support (1-2-5-10 items) [37, 38]. Using the Likert seven-point scoring method, 1 ~ 7 points, the higher the total score, the higher the level of social support. According to the score, perceived social support was divided into high (61 ~ 84), medium (37 ~ 60), and low (12 ~ 36). The scale has been widely used among college students and demonstrated good reliability [39]. The Cronbach’s alpha of the total scale was 0.92 in Zimet et al.,’s study and 0.955 in this study, and the Cronbach’s alpha of the three dimensions was 0.901, 0.927, and 0.866, respectively.

#### Mindfulness attention awareness scale (MAAS)

The scale was compiled by Brown and Ryan and translated into Chinese by Deng et al., including 15 items, such as individual sensation, emotion, cognition, and physiological state [40, 41]. Using the Likert six-point scoring method, ‘1’ represents ‘almost always’, ‘2’ represents ‘very frequent’, ‘3’ represents ‘more frequent’, ‘4’ represents ‘less frequent’, ‘5’ represents ‘very few’, and ‘6’ represents ‘rarely’. According to the scores, the mindfulness level was divided into poor (< 40), medium (41 ~ 65), and good (66 ~ 90). The scale has been widely used among college students and demonstrated good reliability [42]. The Cronbach’s alpha of the scale was 0.78 in Yüksel and Yılmaz’s study and 0.955 in this study.

### Data screening

A total of 256 questionnaires were collected in this study. When sorting out the data, 17 questionnaires were removed because these questionnaires took less than 5 minutes to respond, based on the average filling time of the questionnaires in the pilot study. In addition, 12 questionnaires were excluded in this study because the scores of items in each scale tended to be consistent, which could not reflect the characteristics of individuals. Therefore, these low-quality questionnaires were removed, and finally 227 valid questionnaires were retained.

### Statistical methods

Spss 26.0 and Amos 24.0 were used for data analysis and structural equation model verification. Measurement data are in line with normal distribution, using mean ± standard deviation; enumeration data (such as gender and grade) are described in terms of frequency and percentage. Descriptive statistics were performed on the relevant data using an independent sample t-test and one-way analysis of variance. Pearson correlation analysis was used to test the correlation among psychological distress, social support, mindfulness, and academic self-efficacy (*P* < 0.05). A structural equation model was established based on the result of Pearson correlation analysis. Amos 24.0 was used to establish a structural equation model to analyze and verify the effect of psychological distress on academic self-efficacy under the mediation of social support and mindfulness. Then the Bootstrap method (1000 times) was used to test the mediating effect (95% CI, *P* < 0.05), and the path was analyzed and verified. The approximate root means square error (RMSEA), relative chi-square/degree of freedom (CMIN / DF), comparative fit index (CFI), goodness of fit index (GFI), normal fit index (NFI), incremental fit index (IFI) and Tucker Lewis index (TLI) of the model performed well, indicating that the model fit was good, so the model was not modified in this study.

### Ethical considerations

This study protocol is in line with the ‘Declaration of Helsinki’ and has been approved by the ethical committee of the School of Nursing of Yangzhou University (Ethical number: YZUHL20220029). All questionnaires were filled out voluntarily, anonymously and confidentially. Only investigators have the right to view all information of the questionnaires. In addition, students participating in this study will not receive any awards after completing the questionnaires.

## Results

### General characteristics

Demographic data indicate that the majority of participants in the study were female (192, 84.58%); most are 20 ~ 22 years old; most students come from cities or towns (138, 60.78%); most students have a monthly household income of more than 3000 CNY (163, 71.76%); about half of the students had a moderate level of preference for nursing (117, 51.5%).

### Correlation analysis

All scores of scales are shown in Table 1. Correlation analysis between sample characteristics and academic self-efficacy showed that students’ family location (*F* = 5.027, *P* = 0.007) and academic performance (*F* = 4.442, *P* = 0.005) were significantly correlated with academic self-efficacy. The results of the Pearson correlation analysis showed that there was a significant negative correlation between academic self-efficacy and anxiety and depression, and a significant positive correlation between academic self-efficacy and social support and mindfulness. Anxiety and depression have a significant negative correlation with social support and mindfulness, and there is a significant positive correlation between social support and mindfulness. (see Table 1)

**Table 1:** Means, standard deviations and correlations of all the measures(N = 227)

	Score (mean ± SD)	1	2	3	4	5	6	7	8	9	10
1. Anxiety	4.60 ± 4.271	−									
2. Depression	5.30 ± 5.513	0.778**	−								
3. Social support	60.78 ± 12.476	−0.250**	−0.279**	−							
4. Family support	20.44 ± 4.641	−0.250**	−0.266**	0.927**	−						
5. Friend support	20.59 ± 4.374	−0.245**	−0.260**	0.940**	0.800**	−					
6. Other support	19.74 ± 4.344	−0.205**	−0.257**	0.935**	0.789**	0.837**	−				
7. Mindfulness	62.97 ± 11.581	−0.382**	−0.390**	0.393**	0.387**	0.378**	0.334**	−			
8. Academic self-efficacy	74.42 ± 10.167	−0.257**	−0.287**	0.344**	0.337**	0.343**	0.283**	0.376**	−		
9. Self-efficacy of learning	37.72 ± 6.309	−0.241**	−0.240**	0.293**	0.276**	0.295**	0.249**	0.344**	0.952**	−	
10. Self-efficacy of learning behaviour	36.70 ± 4.583	−0.239**	−0.307**	0.361**	0.368**	0.356**	0.284**	0.360**	0.907**	0.736**	−

Note: *P < 0.05, **P < 0.01

### Multiple mediation analysis

**Fig. 2:**
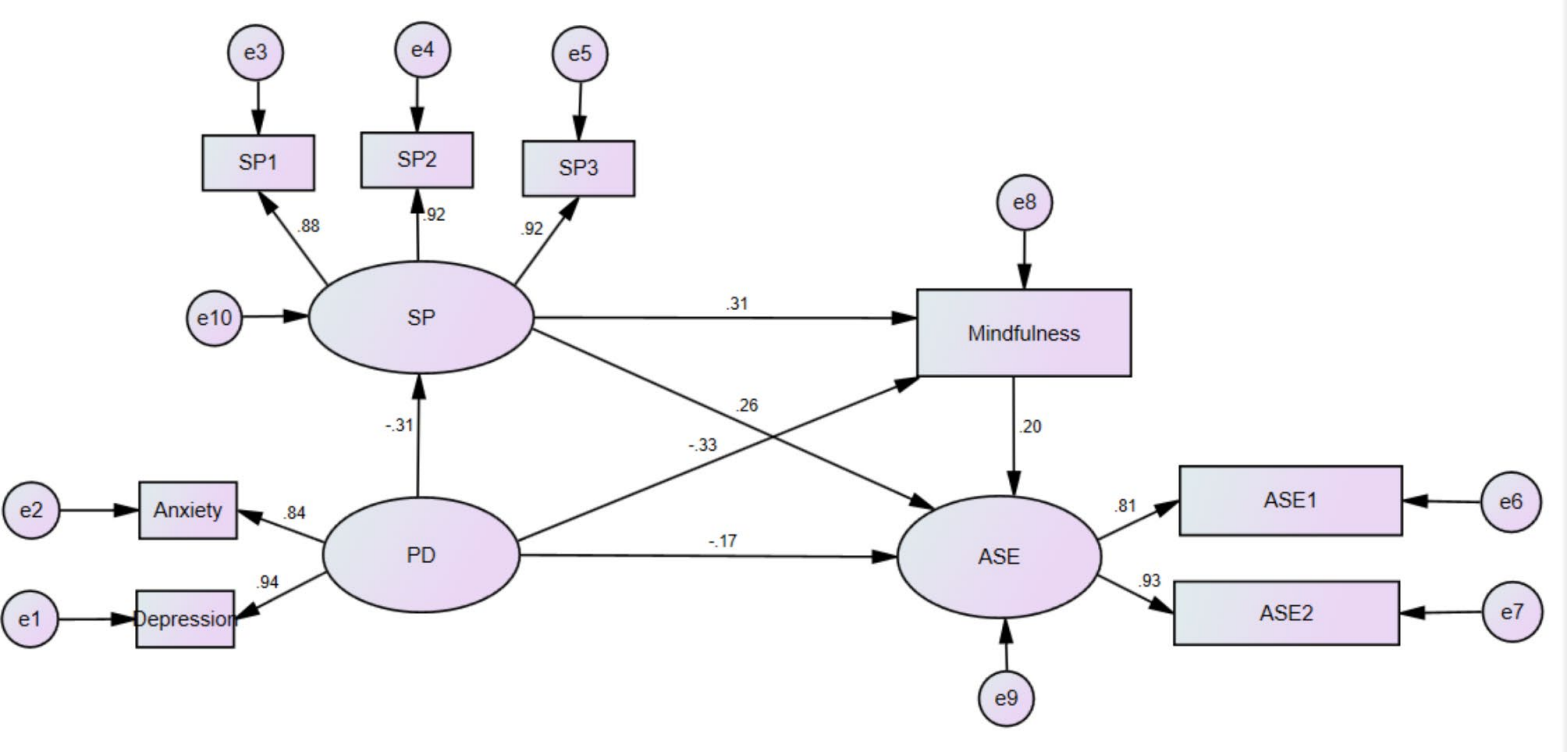
A multiple mediation model of social support and mindfulness mediating psychological distress and academic self-efficacy. Notes: SP = social support; SP1 = family support; SP2 = friend support; SP3 = other support; PD = psychological distress; ASE = academic self-efficacy; ASE1 = Self-efficacy of learning; ASE2 = Self-efficacy of learning behaviour

Combined with the results of correlation analysis, the structural equation model is constructed and the effect of mediating variables in the path and the fitting effect of the overall model is constructed. Randomly select 1000 samples to reduce the incidence of type I errors. According to the previous research hypothesis, nursing undergraduates’ psychological distress (two potential variables: anxiety and depression) was used as a predictor variable in the modelling process; social support (three potential variables: family support, friend support, and other support) and mindfulness were mediating variables; the multiple mediating effect structural equation models was established with academic self-efficacy (two potential variables: self-efficacy of learning ability and self-efficacy learning behaviour) as the effect variable (see Fig. 1). Using Amos 24.0 to verify the action path of the model, the model fitness indexes of the structural equation model meet the standard (See Table 2; Fig. 2), and the results show that the model fits well (CMIN/DF = 1.404, RMSEA = 0.042, GFI = 0.977, IFI = 0.977, TLI = 0.954, CFI = 0.975, NFI = 0.923).

### Total effect, direct effect, and intermediary effect

The direct effect of psychological distress on academic self-efficacy was significant (*β* = −0.168, *P* = 0.026), accounting for 56% of the total effect (*β* = 0.3) Social support partially mediated between psychological distress and academic self-efficacy (M1 = − 0.064, *P* = 0.002). Mindfulness partially mediated between psychological distress and academic self-efficacy (M2 = − 0.053, *P* = 0.019). Social support and mindfulness play a chain mediating role between psychological distress and academic self-efficacy (M3 = − 0.015, *p* = 0.018), and social support can positively predict mindfulness (*β* = 0.311, *P* = 0.001). It indicated that social support and mindfulness played multiple mediating roles in the relationship between psychological distress and academic self-efficacy. The total indirect effect is − 0.132, accounting for 44% of the total effect. M1 accounted for 48.48% of the total indirect effect, M2 accounted for 40.15% of the total indirect effect, and M3 accounted for 11.37% of the total indirect effect. (See Tables 3 and 4)

**Table 2:** Model fit index criteria and fitting results

Index	CMIN/DF	RMSEA	GFI	IFI	TLI	CFI	NFI
Criteria	< 3.000	< 0.080	≥ 0.900	≥ 0.900	≥ 0.900	≥ 0.900	≥ 0.900
Result	1.404	0.042	0.977	0.977	0.954	0.975	0.923

**Table 3:** Standardized Regression Weights

	Estimate	Lower	Upper	*P*
SP<---PD	−0.305	−0.45	−0.141	0.003
Mindfulness<---SP	0.311	0.172	0.445	0.001
Mindfulness<---PD	−0.33	−0.468	−0.171	0.002
ASE<---PD	−0.168	−0.303	−0.017	0.026
ASE<---SP	0.265	0.081	0.447	0.003
ASE<---Mindfulness	0.202	0.011	0.364	0.029
Depression<---PD	0.943	0.829	1.072	0.002
Anxiety<---PD	0.839	0.705	0.942	0.004
SP1<---SP	0.878	0.819	0.919	0.007
SP2<---SP	0.92	0.875	0.955	0.006
SP3<---SP	0.917	0.876	0.952	0.004
ASE1<---ASE	0.806	0.645	0.929	0.003
ASE2<---ASE	0.928	0.775	1.093	0.003

Notes: SP = social support; SP1 = family support; SP2 = friend support; SP3 = other support; PD = psychological distress; ASE = academic self-efficacy; ASE1 = self-efficacy of learning; ASE2 = self-efficacy of learning behaviour.

**Table 4:** Mediation and multiple mediations of social support and mindfulness

Parameter	Estimate	Lower	Upper	*P*
M1: Psychology distress—>Social support—>Academic self-efficacy	−0.064	−0.137	−0.018	0.002
M2: Psychology distress—>Mindfulness—>Academic self-efficacy	−0.053	−0.131	−0.006	0.019
M3: Psychology distress—>Social support—>Mindfulness—>Academic self-efficacy	−0.015	−0.042	−0.002	0.018
M4 = M1 + M2 + M3	−0.132	−0.232	−0.044	0.003

Notes: M1:Mediation of social support; M2: Mediation of mindfulness; M3: Chain mediation of social support and mindfulness; M4: Total indirect effect.

## Discussion

Based on the multi-factor theory of psychological stress, this study investigated the status of academic self-efficacy and its related influencing factors (psychological distress, social support and mindfulness) of nursing undergraduates after COVID-19 and explored the correlation between these variables. Based on the results of correlation analysis, we constructed a structural equation model of academic self-efficacy to explore the mediating role of social support and mindfulness in the impact of psychological distress on academic self-efficacy. This study proves that psychological distress affects the academic self-efficacy of nursing undergraduates through social support and mindfulness. This structural equation model of academic self-efficacy includes three paths: the single mediating role of social support, the single mediating role of mindfulness, and the chain mediating role of social support and mindfulness. Among them, the social support path has the greatest effect value. Our study is the first to explore the current status of academic self-efficacy, its influencing factors and their relationships based on the multi-factor theory of psychological stress. It is helpful for nursing educators and managers to further understand the mechanism of psychological distress affecting the academic self-efficacy of nursing undergraduates and to better reduce the impact of psychological distress on students’ academic self-efficacy.

The study found that the academic self-efficacy of nursing undergraduates was at a good level (74.42 ± 10.167) compared to the median total score. The level of academic self-efficacy in our study is higher than that of Chen et al. The research object of Chen et al. is nursing students who are in the stage of the COVID-19 pandemic and during school isolation. At this stage, students’ normal learning and clinical practice are interrupted, and online learning lacks an effective supervision mechanism, which will affect students’ learning consciousness and academic self-efficacy. Students are vulnerable to external interference, which reduces learning efficiency, enthusiasm, confidence and academic self-efficacy [9]. Nearly half of the students reported different levels of anxiety (46.6%) and generally did not meet the clinical diagnostic criteria for anxiety (4.60 ± 4.271). Compared with the level of anxiety among nursing undergraduates during the COVID-19 pandemic (55.9%), which has been alleviated after COVID-19. The average score of depression (5.30 ± 5.513) had reached the clinical diagnostic criteria for mild depression. Among them, 44.8% of nursing undergraduates reported that they had depressive symptoms, which was also lower than that during the COVID-19 outbreak (48.14%) [16]. After the COVID-19 pandemic, nearly half of nursing undergraduates still have anxiety and depression, which may be related to the following reasons. First of all, the normalization of COVID-19 still causes anxiety and depression among students. Schools in some areas have set up strict school epidemic prevention management measures, such as restricting students from leaving school, maintaining social distancing, and reducing the frequency of recreational activities [43, 44]. These protective measures hinder the normal social communication and interpersonal development of students, which can easily lead to depression. Secondly, no matter where and when, nursing undergraduates have heavy learning tasks [45, 46]. Coupled with their lack of psychological maturity and weak adaptability, they are prone to depression or anxiety under the influence of various pressures [47–49]. In addition, our study found that nursing undergraduates had a higher level of social support (60.78 ± 12.476), and perceived the most support from family and friends, which was consistent with Mai’s results. Under the normalization of COVID-19, students’ mindfulness is above the middle level (62.97 ± 11.581).

Correlation analysis showed that there was a significant negative correlation between academic self-efficacy and psychological distress (*P* < 0.05). The higher the degree of psychological distress, the lower the academic self-efficacy [20]. Higher levels of psychological distress (anxiety and depression) will reduce students’ enthusiasm and confidence in learning, make students appear distracted and reduce learning efficiency. These can affect students’ academic self-efficacy and thus prevent them from achieving their learning goals. Educators should pay attention to the impact of psychological distress on students’ academic self-efficacy.

Additionally, academic self-efficacy was positively correlated with mindfulness and social support (*P* < 0.05). Psychological distress was negatively correlated with mindfulness and social support (*P* < 0.05). Based on the significant correlation between the above variables, we constructed a structural equation model of the influencing factors of academic self-efficacy and verified the three hypotheses proposed before the study.

Firstly, social support was a mediating variable between psychological distress and academic self-efficacy in nursing undergraduates, which confirmed H1. This suggests that nursing undergraduates with lower levels of social support are more likely to affect their academic self-efficacy when their psychological distress is high. According to the theory of social support, the internal and external material or emotional support obtained by individuals can improve the psychological distress of anxiety and depression, thereby enhancing confidence and belief and promoting success [50]. The higher the level of perceived social support, the lower the threat of emotional reactions such as anxiety and fear, and the higher the confidence of individuals to cope with difficulties, the higher the level of self-efficacy, which is conducive to achieving their own goals [51, 52]. Studies have shown that social support plays an important role in improving students’ academic self-efficacy [53]. Therefore, nursing educators can start by improving the social support of nursing undergraduates. By screening students with weak social support, nursing educators can provide students with psychological support and fully mobilize students’ family support and friend support system, enhancing students’ psychological resilience and learning enthusiasm and confidence, so as to alleviate the impact of students’ psychological distress on their academic self-efficacy.

Second, mindfulness was a mediating variable between psychological distress and academic self-efficacy in nursing undergraduates, which confirmed H2. This suggests that nursing undergraduates with a lower level of mindfulness are more likely to affect their academic self-efficacy when they have a higher level of psychological distress. The coping model of mindfulness believes that mindfulness can help individuals re-evaluate negative or threatening events, alleviate psychological distress caused by stress, anxiety, and depression, help individuals regain self-confidence, and enhance the ability to adapt to and respond to various environmental changes [26]. Mindfulness is beneficial to relieve individual psychological distress and improve adaptability and confidence, so as to improve self-efficacy and ultimately achieve goals. Several studies have demonstrated the effects of mindfulness based relaxation training and meditation programs on reducing perceived stress, anxiety, depression and improving the self-efficacy of nursing students [54, 55]. This enlightening nursing educators can improve students’ mindfulness level by applying mindfulness-based stress reduction training or mindfulness meditation, improve students’ cognition of their physical and mental conditions, relieve psychological distress, positively affirm their comprehensive ability, enhance their confidence, and alleviate the influence of psychological distress on academic self-efficacy.

Finally, we tested the chain mediating effect (H3) of social support and mindfulness to alleviate the effect of psychological distress on academic self-efficacy. Several studies have shown a significant positive relationship between social support and mindfulness. The multi-factor theory of psychological stress believes that when faced with stressors, individuals with more social support can pay more attention to their own physical and mental status and focus on the present, thereby reducing uncertainty, anxiety and worry, and ultimately helping individuals cope with stressors in a positive way [56, 57]. In summary, it is suggested that nursing educators should focus on improving students’ social support and mindfulness, so as to relieve students’ psychological distress caused by academic, clinical practice and employment, and finally enhance their confidence, courage and academic self-efficacy to complete learning goals and achieve ideals. Since the end of the COVID-19 pandemic, students have returned to normal study and life. Their perceived social support increased their mental health and social adaptability [58]. In addition, we found that social support can also help people get rid of anxiety and depression by actively raising the level of mindfulness. Mindfulness helps them focus on the present and adjust their state to adapt to the current learning and life, promotes students to generate more academic self-efficacy, and improves learning enthusiasm and academic performance [57, 59, 60].

Although the COVID-19 has passed and the public has returned to normal life and production, the COVID-19 has also brought a warning to the education community. With the global political situation and climate change, public health and security incidents are common, which is very unfavourable for college students with immature psychological development. How to improve the psychological adaptability and academic self-efficacy of college students before the outbreak of public health events is a problem worthy of in-depth consideration by educators around the world.

In summary, educators need to timely screen and find nursing students with psychological distress-related manifestations, and care about students’ level of social support and mindfulness. For students with less social support, educators and managers should provide timely care and help to improve their level of peer support. In addition, effective psychological interventions such as mindfulness therapy and cognitive behavioural therapy can also be selectively used in student groups. To reduce students’ psychological distress and improve students’ learning enthusiasm and academic self-efficacy, help students to resume normal learning and lay a solid foundation for future nursing work.

## Limitation

First, the study was conducted at a single university in Jiangsu Province, China, which limits the representativeness of the sample size and the generalizability of the findings. Secondly, the questionnaire was filled out by students independently, and the quality of the data could not be fully guaranteed. Thirdly, only anxiety and depression, the two most common symptoms of psychological distress, were investigated in this study and other variables related to psychological distress were not explored. Finally, this study only explored the mediating role of social support and mindfulness in the influence of psychological distress on academic self-efficacy among nursing undergraduates. Potential influencing variables such as stress and coping style may also affect students’ academic self-efficacy, which needs to be further verified.

## Conclusion

This study can be seen as the evidence of the direct and indirect effects of psychological distress, social support, and mindfulness on academic self-efficacy among nursing undergraduates. The psychological distress of nursing undergraduates with higher levels of social support and mindfulness may have less impact on their academic self-efficacy. Therefore, educators can relieve the physical and mental stress of nursing undergraduates and promote students to focus on their own thoughts and learning development by increasing students’ peer support and implementing mindfulness related courses.

## Data Availability

The datasets used during this study are available from the corresponding author on reasonable request.
